# Brief historical account of autism in Peru

**DOI:** 10.17843/rpmesp.2024.412.13358

**Published:** 2024-04-08

**Authors:** Marilia Baquerizo-Sedano, Joë Lucero, Alvaro Taype-Rondan

**Affiliations:** 1 University of Burgos, Health Sciences Faculty, Burgos, Spain. University of Burgos University of Burgos Health Sciences Faculty Burgos Spain; 2 Turing: science, technology and neurodiversity, Lima, Peru. Turing: science, technology and neurodiversity Lima Peru; 3 Universidad Peruana Cayetano Heredia, Facultad de Psicología, Lima, Peru. Universidad Peruana Cayetano Heredia Universidad Peruana Cayetano Heredia Facultad de Psicología Lima Peru; 4 Research Unit for the Generation and Synthesis of Health Evidence, Vice-Rectorate for Research, San Ignacio de Loyola University, Lima, Peru. San Ignacio de Loyola University Research Unit for the Generation and Synthesis of Health Evidence Vice-Rectorate for Research San Ignacio de Loyola University Lima Peru; 5 EviSalud - Evidencias en Salud, Lima, Peru. EviSalud - Evidencias en Salud Lima Peru

**Keywords:** Autism, Peru, Autism Spectrum Disorders, Disability

## Abstract

We present a historical account of autism in Peru. Currently, the term “autism spectrum conditions”, from the neurodiversity paradigm, is used to describe neurodevelopmental disorders characterized by persistent difficulties in communication, social interaction, and restricted and repetitive behaviors and interests. In Peru, the scientific study of nervous and mental diseases began around 1920 and although the diagnosis of “childhood autism” was proposed in 1959, it only began to spread in the 1980s. Although significant advances were made in the 21st century, Peru still faces many challenges in addressing autism.

## INTRODUCTION

Autism spectrum conditions (ASC), from the neurodiversity paradigm, or autism spectrum disorders (ASD), in psychodiagnostics manuals, are very heterogeneous neurodevelopmental conditions characterized by a cognitive phenotype ^1^ underlying a specific combination of: a) persistent difficulties in communication and social interaction, and b) presence of restrictive and repetitive patterns of behavior, interests or activities ^2^. A recent systematic review reported a worldwide ASC prevalence rate of about 1% ^3^.

The term “autism” was introduced in 1911 by Eugen Bleuler to refer to withdrawal into the inner world, a symptom of schizophrenia. Those who first described autism as a clinical picture were Grunya Suckareva in the article entitled *Die schizoiden Psychopathien im Kindesalter* (1926) ^4^; Hans Asperger, who spoke of children with a certain pattern of behavior at the University Hospital of Vienna (1938) and later published the German article *Die Autistischen Psychopathen im Kindesalter* (1944) ^5^; and Leo Kanner, in the English article, Autistic disturbances of affective contact (1943) ^6^. The latter, was the most widespread article in the following decades since it was published in English, although autism was still perceived as a condition very close to that of “infantile schizophrenia”. It is still in 1980, in the Diagnostic and Statistical Manual of Mental Disorders III (DSM III), that “infantile autism” is incorporated as a specific diagnostic category.

Autism was considered to be a spectrum in the DSM IV (1994), and “autism spectrum disorders” were placed under “pervasive developmental disorders”. At that time, five subtypes of autism were included: autistic disorder, Asperger syndrome, childhood disintegrative disorder, pervasive developmental disorder not otherwise specified (PDD not otherwise specified), and Rett syndrome. Subtypes were no longer considered in the DSM 5 (2013), and with a more quantitative-dimensional, rather than qualitative-categorical approach, the autism spectrum was organized into levels of support: level 1 (needs support), level 2 (needs notable support) and level 3 (needs very notable support).

The history of ASC shows how the dominant theories from each era influenced the terms used in descriptions, diagnostic criteria, differential diagnosis, causal hypotheses and therapeutic interventions.

Understanding the history of autism in Peru may be useful to identify the factors that influenced its evolution over time, which may allow a better understanding of current challenges. Therefore, this article presents a brief historical account of autism in Peru.

## METHODOLOGY

We used a documentary research methodology. In order to gather sources, searches were carried out between January and July 2022.

We searched Google and Google Scholar using related key terms, such as [autism Peru], [childhood schizophrenia Peru], among others. This strategy allowed the recollection of relevant sources, both scientific articles, governmental reports and institutional documents.

In addition, we reviewed the catalogs of specialized libraries (library of the Victor Larco Herrera Hospital and the Universidad Peruana Cayetano Heredia), which include titles such as *Revista de Neuro-Psiquiatría* (founded in 1938). From these catalogs, we selected publications that included articles with the terms: [dementia praecox], [childhood schizophrenia] and [childhood autism]; as well as secondary sources, such as twentieth century publications on the history of psychiatry and psychology in Peru.

Each source we collected was analyzed and organized for the writing of the article and obtaining citations and references.

## EARLY YEARS OF THE SCIENTIFIC STUDY OF NERVOUS AND MENTAL ILLNESSES

The scientific study of nervous and mental illnesses in Peru began in 1915, thanks to Hermilio Valdizán, a Peruvian physician particularly interested in childhood ^7^. He trained for four years, from 1911 to 1914, in European clinics, spending most of his time in Rome, under the direction of Sante de Sanctis, a pioneer of child psychiatry in the world ^8^.

After his trip in 1916, Valdizán organized the chair of nervous and mental illnesses in the medical school of the Universidad Nacional Mayor de San Marcos (UNMSM), the oldest higher education center in America, and inaugurated in 1918 the Colonia de la Magdalena Asylum, which in 1930 was renamed the Victor Larco Herrera Hospital. From these two spaces, and together with Honorio Delgado, he began to study “abnormal childhood” and proposed preventive programs with pedagogical orientation ^8^.

**Figure 1:**
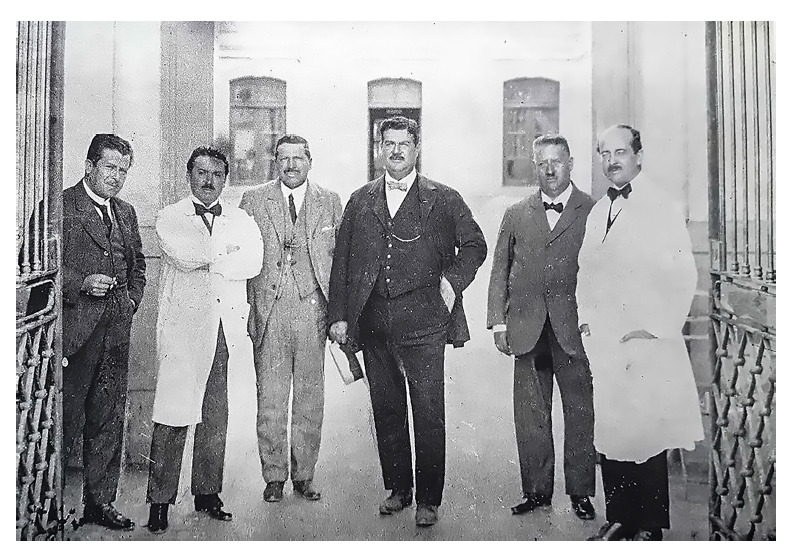
From left to right: Hermilio Valdizán, Baltazar Caravedo Prado, Wenceslao Mayorga, Víctor Larco Herrera, Estanislao Pardo Figueroa and Sebastián Lorente Patrón. Source: Sociedad de Beneficencia Pública de Lima (1920). Victor Larco Herrera Hospital, formerly Colonia de la Magdalena Asylum.

Figure 1 shows a photograph of Valdizán, Caravedo (an outstanding psychiatrist), Larco Herrera (a philanthropic businessman who funded part of the construction and equipment of the Colonia de la Magdalena Asylum) and the doctors who worked there. After Valdizán’s untimely death in 1929, Baltazar Caravedo took over the direction of the Colonia de la Magdalena Asylum and in 1932 created the first child neuropsychiatry office; years later, in 1938, the “Ward for psychopathic and abnormal children” was inaugurated, under the direction of Carlos Krumdieck ^9^.

The memoirs of the Victor Larco Herrera Hospital from 1938 include the “Classification of psychiatric problems in children” elaborated by Caravedo ^9^. In this classification, “dementia praecox” can be found within the Psychosis category, a clinical picture defined by Emil Kraepelin in 1896. A few years later, the term “infantile schizophrenia”, as proposed by Eugen Bleuler in 1911, started to be used. In 1941, Luis Guerra founded the National Pedagogical Institute, which included a Department of Special Pedagogy ^10^.

To our knowledge, the first article that refers to a case of “infantile schizophrenia” in Peru, was that of Luis Guerra in 1944, which presents the details of the medical history of a 12-year-old boy and his diagnosis is discussed at length ^11^. Given the appearance of several associated terms and the difficulty to clearly differentiate the medical pictures, a strong debate regarding child psychopathology started in the country in the 1940s.

At that time, some Peruvian psychiatrists, such as Francisco Alarco (1945), promoted the concept that mental disorders could have multiple causes, which implies taking into account several factors regarding prevention, and suggests an integral approach when it comes to treatment ^(^^12^. Alarco encouraged his colleagues to have an attitude free of the prejudices of the prevailing theories, particularly regarding childhood. However, the psychoanalytic theory had a significant influence on the conception of cases; for example, in 1955, Niza Chiock de Majluf published the article “Mother-child relationship in childhood schizophrenia” ^13^.

Although in the 1940s and 1950s interventions were driven by psychoanalysis, educationally oriented interventions as well as a valuable approach aimed at optimizing children’s development were also proposed. In 1946, Emiliano Pisculich and Enriqueta Arroyo wrote the essay “The education of abnormal children”, and focused on the importance of a special education focused on vocational training, on the cultivation of a “faculty for something”. Both authors belonged to the Department of Special Paidology directed by Luis Guerra ^10^.

During the 1950s, Peruvian psychiatrists were aware of the most important foreign publications and considered the approaches of Leo Kanner, a pioneer in the description of the clinical picture of autism in the United States ^14^. However, autism was still referred to as a symptom and not as a clinical picture. In 1959, Emilio Majluf presented the case of a four-year-old boy who had “difficulties in affective contact, developmental delay (particularly language), and behavioral and habit disorders, which caused deep anxiety” ^15^.

Majluf refers that the diagnosis of “early infantile autism” was proposed, but faced with “the uncertainty of its clinical delimitation and the difficulties to specify its etiology”; it was preferred to consider the case as an “infantile schizomorphic syndrome”, which would be the product of psychological influence by the mother in the early years and the disorganization of the family environment, among other factors, “being difficult to prove the presence of constitutional factors or of a conditioning brain pathology” ^15^. In the same year, Majluf published an article on the diagnosis and treatment of behavioral disorders in children ^16^, in which he points out the main problems in categorization, and highlights the inadequacy of the classification systems of that time.

## EARLY YEARS OF “INFANTILE AUTISM” AS A CLINICAL CONDITION

By the 1960s, 45% of the population in Peru was under 15 years of age, and about 50% of the consultations in neuropsychiatric services in Lima were of children and adolescents ^8^. Verna Alva León took special interest in children and adolescents, and in 1973 published her doctoral thesis “*La psiquiatría infantil en el Perú. Aspectos asistenciales, epidemiológicos y de organización en Lima Metropolitana*” (Child psychiatry in Peru. Aspects of care, epidemiology and organization in Metropolitan Lima.) ^8^.

In the 1970s there was already talk of “Infantile Autism” as a clinical condition, but unfortunately, care was still deficient and was provided exclusively in the capital, where the number of hospitals increased and centers run by nuns were created. Lily Mayo, a Peruvian teacher, says that in 1975 she visited a religious center where she saw autistic children and adults locked in cages, one of them with a sign that read: “don't come close because I bite”; she also saw naked children tied up with their shirts as punishment for hitting their heads or biting themselves ^17^.

This experience led Mayo to create in Lima, in 1979, the Ann Sullivan Center of Peru, where educational programs for people with autism and other disabilities began to be carried out. Applied behavioral analysis was already known in Peru in the 1980s, therefore, the center initially followed this approach and considered a curriculum with emphasis on the development of autonomy, communication and skills for work and community performance ^18^, which later became known as the “functional-natural curriculum”. In 1986, the center began to conduct research along different lines and with much outside collaboration ^18^.

The addition of “infantile autism” as a specific category in the DSM III (1980) facilitated diagnosis and more structured interventions, both in private and public Peruvian centers. A study that included 22 children with “infantile autism” between 1982 and 1986 in a public hospital in Lima reported that 50% had “fair or good” success, and that the factors associated with success were, among others, intellectual and social quotient ^19^.

As in other countries during those years ^20^, there was an attempt in Peru to identify the factors associated with treatment success and to typify cases of autism according to these factors, which were highly variable. In the 1980s, behaviorism was in vogue in Peru, but cognitivism was gradually arriving, especially in the educational field ^10^.

Psychometric studies began to consider more variables ^21^, studies on early stimulation ^22^ and the diagnosis and treatment of “exceptional children” ^23^ were published. In addition, several studies compared social groups differentiated by socioeconomic class or place of residence. These lines of research had a correspondence with the extension of programs to combat child malnutrition in localities of poverty or extreme poverty (Wawa Wasi National Program), and the increase of early education and special education centers at the national level.

In the 1990s there were more professional training opportunities, therefore, the treatment of people with autism in Peru began to be carried out by psychologists, special educators and medical technologists (occupational, physical and speech therapists), who introduced new programs. Bondy and Frost in 1993 ^24^ introduced, for example, the Picture Exchange Communication System-PECS, an alternative/augmentative communication system that focuses on the exchange of pictures. Between 1993 and 2000, the Ministry of Education, with technical assistance from UNESCO, developed the Project for the Integration of Children with Special Educational Needs into Regular Schools, which included 234 educational centers and 768 children ^25^.

## PROGRESS IN THE 21ST CENTURY

The broader consideration of autism as an “autism spectrum disorder” in the DSM-IV (1994) coincided with the development of models with greater explanatory scope, such as the theory of mental blindness ^26^, which led to an increase in the number of diagnoses and knowledge about the condition worldwide, and also in Peru, although on a different scale. To date, there are still no epidemiological studies on autism in Peru, and there is only the National Registry of Persons with Disabilities under the National Council for the Integration of Persons with Disabilities (CONADIS), which according to information requested from the National Disability Observatory (https://observatorio.conadisperu.gob.pe) reports four registrations of persons with autism in 2001, 836 in 2011 and 8835 in 2021 ^27^.

On the other hand, in 1999, the first parents’ association in the country was created and was called *Asociación de Padres y Amigos de Personas con Autismo* (ASPAU - Peru). In the 2010s, in Lima, public health centers began to provide specialized care for autism, while private centers for people with this condition multiplied, although they focused mainly on children and adolescents, not adults. 

The DSM-5 was published in 2013 and it follows the biopsychosocial model; this model contrasts with the biomedical one because it considers that disability does not only depend on the conditions of the person, but also on the support that may or may not be provided by the environment. This change of approach coincides with the dissemination of the neurodiversity paradigm in Peru ^28^, which was born in 1998 in the United States and arrived in Latin America almost a decade later. This paradigm considers that ASC are part of the natural variation of the human species, at the cognitive and cerebral level, and just as it suggests difficulties in some areas, it is associated with strengths in others.

In 2014, Law No. 30150, Law for the Protection of Persons with Autism Spectrum Disorder ^29^ was enacted, which aimed to “establish a legal regime that promotes early detection and diagnosis, early intervention, health protection, comprehensive education, professional training and labor and social insertion of persons with autism spectrum disorder”. In 2019, the first National Plan for people with Autism Spectrum Disorder (2019 - 2021) was formulated ^30^.

In recent years, the autistic collective has become more socially visible, several organizations carry out visibility events on representative days and some media disseminate related news; likewise, there have been many advances in interdisciplinary intervention that allow improving the quality of life of people with ASC; however, it is not enough to improve the recognition and defense of the rights of people with ASC. 

**Figure 2:**
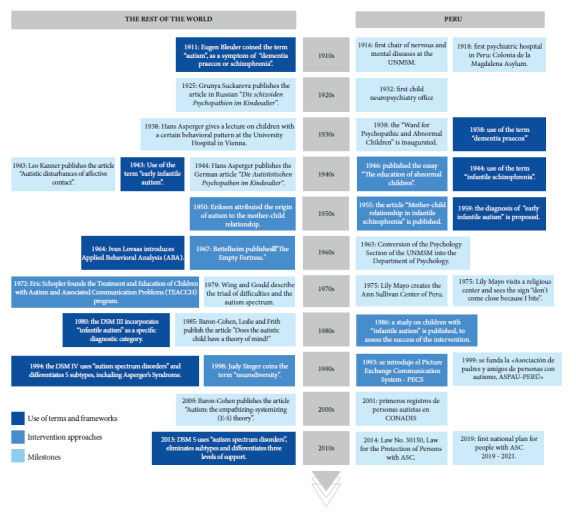
Timeline of autism in Peru, in contrast with the rest of the world.

Figure 2 shows a timeline that summarizes the history of autism in Peru; in order to demonstrate the contrast, this figure includes information from Peru and the rest of the world related the use of terms, intervention approaches and milestones, in the decade of 1910 to 2010. The variation of terms associated with autism over time, from “dementia praecox” to “infantile autism” and “autism spectrum disorders”, are related to the perception of the condition and its approach.

On the other hand, the constant gap between the progress of knowledge on autism at the international level and its integration in the Peruvian context stands out. This is reflected in the disparity in the availability of services, access to specialized health care and education, and research. This contrast highlights the need for a renewed focus on the development and implementation of specific strategies to address the needs of people with ASC in the Peruvian context.

Despite significant advances in early detection, diagnosis, and intervention since the 1980s and particularly in the 21st century, there are still considerable challenges in Peru in terms of a comprehensive understanding of ASC, the availability of and access to specialized services, and the integration of these individuals into society. Currently, Peru still faces many challenges about understanding ASC, its diagnosis (especially early diagnosis), epidemiology, legislation, management and research. Understanding and reflecting on the history of autism in Peru allows us to have a frame of reference to face these challenges.

ASC is a global challenge, its prevalence is close to 1%, and its study has become increasingly important worldwide. However, there has been a notable lack of specific historical research on autism in Peru. This article arises as a response to this research gap, highlighting the evolution of the terms associated with autism over time, from the denomination of “dementia praecox” in 1938, through “infantile schizophrenia” in 1944, to “infantile autism” in 1959, and the subsequent extension of the diagnosis in the 1980s, as well as the advances and challenges in the 21st century. These findings are not only relevant from a historical perspective, but also have important implications for public awareness, health policy development and implementation of specialized services to effectively address autism in the specific context of Peru.
