# Supplementary material for: Brief historical account of autism in Peru
Source: Rev Peru Med Exp Salud Publica. 2024 Apr 8;41(2):214–9. doi: 10.17843/rpmesp.2024.412.13358 (PMC11300681; doi:10.17843/rpmesp.2024.412.13358)
Supplement: Translation

# Translation: Breve recuento histórico del autismo en Perú

**Authors:** Marilia Baquerizo-Sedano, Joë Lucero, Alvaro Taype-Rondan

**Affiliations:** 1 Universidad de Burgos, Facultad de Ciencias de la Salud, Burgos, España.; 2 Turing: ciencia, tecnología y neurodiversidad, Lima, Perú.; 3 Universidad Peruana Cayetano Heredia, Facultad de Psicología, Lima, Perú.; 4 Unidad de Investigación para la Generación y Síntesis de Evidencias en Salud, Vicerrectorado de Investigación, Universidad San Ignacio de Loyola, Lima, Perú.; 5 EviSalud - Evidencias en Salud, Lima, Perú.

**Keywords:** Autismo, Perú, Trastornos del Espectro Autista, Discapacidad

## Abstract

Presentamos un recuento histórico del autismo en el Perú. Actualmente, desde el paradigma de la neurodiversidad, se utiliza el término «condiciones del espectro autista» para describir alteraciones del neurodesarrollo caracterizadas por dificultades persistentes en la comunicación, la interacción social y comportamientos e intereses restringidos y repetitivos. En Perú, el estudio científico de las enfermedades nerviosas y mentales comenzó alrededor de 1920, y aunque el diagnóstico de «autismo infantil» se planteó en 1959, comenzó a extenderse recién en la década de 1980. Si bien en el siglo XXI se presentaron avances significativos, Perú todavía enfrenta muchos retos en el abordaje del autismo.

## INTRODUCCIÓN

Las condiciones del espectro autista (CEA), desde el paradigma de la neurodiversidad, o trastornos del espectro autista (TEA), en los manuales de psicodiagnóstico, son condiciones del neurodesarrollo muy heterogéneas que se caracterizan por un fenotipo cognitivo ^1^ que subyace a una combinación específica de: a) dificultades persistentes en la comunicación y la interacción social, y b) presencia de patrones restrictivos y repetitivos de comportamiento, intereses o actividades ^2^. Una revisión sistemática reciente señala que las CEA tendrían una prevalencia mundial cercana al 1% ^3^.

El término «autismo» fue introducido en 1911 por Eugen Bleuler, para hacer referencia al repliegue en el mundo interior, un síntoma de la esquizofrenia. Quienes primero describieron al autismo como cuadro clínico fueron Grunya Suckareva en el artículo titulado Die schizoiden Psychopathien im Kindesalter (1926) ^4^; Hans Asperger, quien habló de niños con determinado patrón de conducta en el Hospital Universitario de Viena (1938) y luego publicó el artículo en alemán Die Autistischen Psychopathen im Kindesalter (1944) ^5^; y Leo Kanner, en el artículo en inglés Autistic disturbances of affective contact (1943) ^6^. Esta última, por publicarse en inglés, fue la que más se extendió en las décadas siguientes, aunque seguía percibiéndose como un cuadro muy cercano al de «esquizofrenia infantil». Es todavía en 1980, en el Diagnostic and Statistical Manual of Mental Disorders III (DSM III), que se incorpora al «autismo infantil» como categoría de diagnóstico específico.

En el DSM IV (1994) se consideró al autismo como espectro y se colocó a los «trastornos del espectro autista» dentro de los «trastornos generalizados del desarrollo». En ese entonces se incluían cinco subtipos de autismo: el trastorno autista, el síndrome de Asperger, el trastorno desintegrativo infantil, el trastorno generalizado del desarrollo no especificado (TGD no especificado) y el síndrome de Rett. En el DSM 5 (2013) se deja de considerar subtipos, y con un enfoque más cuantitativo-dimensional, que cualitativo-categórico, se organiza el espectro autista en niveles de apoyo: nivel 1 (necesita apoyo), nivel 2 (necesita apoyo notable) y nivel 3 (necesita apoyo muy notable).

La historia de las CEA muestra cómo las teorías dominantes de cada época influyeron en los términos que se utilizaron en las descripciones, los criterios diagnósticos, el diagnóstico diferencial, las hipótesis causales y las intervenciones terapéuticas.

Conocer la historia del autismo en el Perú puede ser útil para identificar los factores que influyeron en su evolución a lo largo del tiempo, lo que puede permitir comprender mejor los desafíos actuales. Por ello, el presente artículo presenta un breve recuento histórico del autismo en el Perú.

## METODOLOGÍA

Se utilizó una metodología de investigación documental. Para el arqueo de fuentes se efectuaron búsquedas entre enero y julio del 2022.

Se realizaron búsquedas en Google y Google Académico, usando términos clave relacionados, como [autismo Perú], [esquizofrenia infantil Perú], entre otros. Esta estrategia permitió recopilar fuentes relevantes, tanto de artículos científicos como de informes gubernamentales y documentos institucionales.

Adicionalmente, se revisaron los catálogos de bibliotecas especializadas (biblioteca del Hospital Víctor Larco Herrera y de la Universidad Peruana Cayetano Heredia), que incluyen títulos como la Revista de Neuro-Psiquiatría (fundada en 1938). En dichos catálogos se revisaron aquellas publicaciones que incluían artículos con los términos: [demencia precoz], [esquizofrenia infantil] y [autismo infantil]; así como fuentes secundarias, como publicaciones del siglo XX sobre la historia de la psiquiatría y de la psicología en Perú.

Cada fuente encontrada en las búsquedas fue analizada y organizada para la redacción del artículo y la obtención de citas y referencias.

## PRIMEROS AÑOS DEL ESTUDIO CIENTÍFICO DE LAS ENFERMEDADES NERVIOSAS Y MENTALES

El estudio científico de las enfermedades nerviosas y mentales empezó a cultivarse en el Perú en 1915, gracias a Hermilio Valdizán, médico peruano especialmente interesado en la infancia ^7^. Él se formó durante cuatro años, de 1911 a 1914, en clínicas europeas, pasando la mayor parte de tiempo en Roma, bajo la dirección de Sante de Sanctis, pionero de la psiquiatría infantil en el mundo ^8^.

Después de su viaje en 1916, Valdizán organizó la cátedra de enfermedades nerviosas y mentales en la facultad de medicina de la Universidad Nacional Mayor de San Marcos (UNMSM), el centro de educación superior más antiguo de América, e inauguró en 1918 el Asilo Colonia de la Magdalena, que en 1930 pasó a llamarse Hospital Víctor Larco Herrera. Desde estos dos espacios, y junto a Honorio Delgado, empezó a estudiar la «infancia anormal», y propuso programas preventivos con orientación pedagógica ^8^.

En la figura 1 presentamos una fotografía de Valdizán, Caravedo (destacado médico psiquiatra), Larco Herrera (empresario filántropo que financió parte de la construcción y equipamiento del Asilo Colonia de la Magdalena) y médicos que trabajaban ahí. Tras el prematuro fallecimiento de Valdizán en 1929, Baltazar Caravedo asumió la dirección del Asilo Colonia de la Magdalena y en 1932 creó el primer consultorio de neuropsiquiatría infantil; años después, en 1938, se inauguró el «Pabellón para niños psicópatas y anormales», a cargo de Carlos Krumdieck ^9^.

**Figura 1:** De izquierda a derecha: Hermilio Valdizán, Baltazar Caravedo Prado, Wenceslao Mayorga, Víctor Larco Herrera, Estanislao Pardo Figueroa y Sebastián Lorente Patrón. Fuente: Sociedad de Beneficencia Pública de Lima (1920). Hospital Víctor Larco Herrera, antes Asilo Colonia de la Magdalena.

En las memorias del Hospital Víctor Larco Herrera del año 1938, se muestra la «Clasificación de los problemas psiquiátricos en los niños» elaborada por Caravedo ^9^. En esta clasificación, dentro de la categoría Psicosis, se observa «demencia precoz», cuadro clínico definido por Emil Kraepelin en 1896. Unos años después, se empezó a hablar de «esquizofrenia infantil», en los términos que planteó Eugen Bleuler en 1911. En 1941, Luis Guerra fundó el Instituto Pedagógico Nacional, donde se estableció un Departamento de Paidología Especial ^10^.

A nuestro conocimiento, el primer artículo encontrado que hace referencia a un caso de «esquizofrenia infantil» en el Perú, fue el de Luis Guerra en 1944, donde se exponen los detalles de la historia clínica de un niño de 12 años, y se discute ampliamente su diagnóstico ^11^. Dada la aparición de varios términos asociados y la dificultad para diferenciar claramente los cuadros, se originó, en la década de 1940, un fuerte debate en el país alrededor de la psicopatología infantil.

Algunos psiquiatras peruanos de aquellos años, como Francisco Alarco (1945), promovieron la aceptación de la etiología múltiple de los trastornos mentales, lo que en prevención implica tomar en cuenta varios factores, y en el tratamiento, un enfoque integral ^12^. Especialmente con relación a la infancia, Alarco animó a sus colegas a tener una actitud libre de los prejuicios propios de las teorías reinantes. Sin embargo, la teoría psicoanalítica influyó notoriamente en la concepción de los casos; por ejemplo, Niza Chiock de Majluf publicó en 1955 el artículo «Relación madre-hijo en la esquizofrenia infantil» ^13^.

Aunque en las décadas de 1940 y 1950 las intervenciones se dieron bajo la sombra del psicoanálisis, también se propusieron intervenciones con orientación educativa y un valioso enfoque orientado a optimizar el desarrollo de los niños. En 1946, Emiliano Pisculich y Enriqueta Arroyo escribieron el ensayo «La educación de los niños anormales», y se centraron en la importancia de una educación especial enfocada en la formación vocacional, en el cultivo de una «facultad para algo». Ambos autores pertenecían al Departamento de Paidología Especial dirigido por Luis Guerra ^10^.

Durante la década de 1950, la psiquiatría peruana estuvo al tanto de las publicaciones foráneas más importantes y se consideró los planteamientos de Leo Kanner, pionero en la descripción del cuadro clínico del autismo en Estados Unidos ^14^. Sin embargo, se seguía hablando del autismo como síntoma y no como cuadro clínico. En 1959, Emilio Majluf expuso el caso de un niño de cuatro años que presentaba «dificultades en el contacto afectivo, retardo en el desarrollo (especialmente en el lenguaje), y trastornos de la conducta y de los hábitos, los cuales causaban una profunda ansiedad» ^15^.

Majluf refiere que se planteó el diagnóstico de «autismo infantil precoz», pero frente a «la incertidumbre de su delimitación clínica y las dificultades para precisar su etiología», se prefirió considerar el caso como un «síndrome esquizomorfo infantil», que sería producto de influencias psicológicas dimanadas por la madre en los primeros años y la desorganización del ambiente familiar, entre otros factores, «siendo difícil probar la presencia de factores constitucionales o de una patología cerebral condicionante» ^15^. El mismo año, Majluf publicó un artículo sobre el diagnóstico y tratamiento de los trastornos del comportamiento en el niño ^16^, donde señala los principales problemas en la categorización, y destaca la insuficiencia de los sistemas de clasificación de aquel entonces.

## PRIMEROS AÑOS DEL «AUTISMO INFANTIL» COMO CUADRO CLÍNICO

Para la década de 1960, el 45% de la población en Perú tenía menos de 15 años, y cerca del 50% de las consultas en servicios neuropsiquiátricos en Lima eran de niños y adolescentes ^8^. Verna Alva León fue quien puso especial interés en los niños y adolescentes, y en 1973 publica su tesis doctoral «La psiquiatría infantil en el Perú. Aspectos asistenciales, epidemiológicos y de organización en Lima Metropolitana» ^8^.

En la década de 1970 ya se hablaba de «Autismo Infantil» como cuadro clínico, pero lamentablemente, la atención aún era deficiente y se daba exclusivamente en la capital, donde aumentó el número de hospitales y se crearon centros a cargo de religiosas. Lily Mayo, una maestra peruana, cuenta que en 1975 visitó un centro religioso en donde vio niños y adultos autistas encerrados en jaulas, y una de ellas con un cartel que decía: «no te acerques porque muerdo»; además vio a niños desnudos atados con sus polos, como castigo por golpearse la cabeza o morderse ^17^.

Esta experiencia llevó a Mayo a crear en Lima, en 1979, el Centro Ann Sullivan del Perú, donde se empezaron a realizar programas educativos para personas con autismo y con otras discapacidades. Como en la década de 1980 ya se conocía en Perú el análisis conductual aplicado, el centro siguió en un inicio este enfoque y consideró un currículo con énfasis en el desarrollo de la autonomía, la comunicación y las habilidades para el desempeño en el trabajo y la comunidad ^18^, que luego pasó a llamarse «currículo funcional-natural». A partir de 1986, el centro empezó a hacer investigación tomando en cuenta diferentes líneas y con mucha colaboración exterior ^18^.

La incorporación de «autismo infantil» como categoría específica en el DSM III (1980) facilitó el diagnóstico y la realización de intervenciones más estructuradas, tanto en centros privados como públicos de Perú. En un estudio que incluyó 22 niños con «autismo infantil», atendidos entre 1982 y 1986 en un hospital público de Lima, se encontró que el 50% tuvo un éxito «regular o bueno», y que los factores asociados al éxito eran, entre otros, el coeficiente intelectual y social ^19^.

Como en otros países ^20^, en esa época se buscaba identificar los factores asociados al éxito del tratamiento y se intentaba tipificar en función a estos factores los casos de autismo, que de hecho eran muy variables entre sí. En la década de 1980, el conductismo estaba en boga en el Perú, pero el cognitivismo iba llegando poco a poco, especialmente en el ámbito educativo ^10^.

Con relación a la investigación, los estudios psicométricos empezaron a considerar más variables ^21^, se publicó sobre estimulación temprana ^22^ y, sobre el diagnóstico y tratamiento de «niños excepcionales» ^23^. Además, se desarrollaron varios estudios que comparaban grupos sociales diferenciados por la clase socioeconómica o el lugar de residencia. Estas líneas de investigación tuvieron una correspondencia con la extensión de programas para combatir la desnutrición infantil en localidades de pobreza o pobreza extrema (Programa Nacional Wawa Wasi), y el aumento de centros de educación inicial y educación especial a nivel nacional.

Dado que en la década de 1990 ya se contaba con más ofertas de formación profesional, el tratamiento de personas con autismo en Perú empezó a estar a cargo de psicólogos, educadores especiales y tecnólogos médicos (terapeutas ocupacionales, físicos y de lenguaje), quienes introdujeron nuevos programas. Bondy y Frost en 1993 ^24^ introdujeron, por ejemplo, el Picture Exchange Communication System-PECS, un sistema alternativo/aumentativo de comunicación que se centra en el intercambio de imágenes. Entre 1993 y 2000, el Ministerio de Educación, y con asistencia técnica de la UNESCO, desarrolló el Proyecto de Integración de niños con necesidades educativas especiales a las escuelas regulares, cuyo reporte señala que participaron 234 centros educativos y 768 niños ^25^.

## AVANCES EN EL SIGLO XXI

La consideración más amplia respecto del autismo como «trastorno del espectro autista» en el DSM-IV (1994) coincidió con el desarrollo de modelos con mayor alcance explicativo, como la teoría de la ceguera mental ^26^, lo cual hizo que el número de diagnósticos y el conocimiento sobre la condición aumente a nivel mundial y también en Perú, aunque a diferente escala. Hasta la actualidad aún no se cuentan con estudios epidemiológicos sobre autismo en Perú, y solo se tiene el Registro Nacional de la Persona con Discapacidad a cargo del Consejo Nacional para la Integración de las Personas con Discapacidad (CONADIS), que según la información solicitada mediante el Observatorio Nacional de la Discapacidad (https://observatorio.conadisperu.gob.pe) reporta cuatro inscripciones de personas con autismo en el año 2001, 836 en el año 2011 y 8835 en el año 2021 ^27^.

Por otro lado, en 1999, se formó la primera asociación de padres en el país denominada Asociación de Padres y Amigos de Personas con Autismo (ASPAU - Perú). En la década de 2010, en Lima, los centros de salud públicos empezaron a brindar atención especializada para autismo, mientras que los centros privados que atendían este tipo de personas se multiplicaron, aunque se enfocaron principalmente en niños y adolescentes, no en adultos.

En el 2013 se publicó el DSM-5, que sigue el modelo biopsicosocial; este modelo contrasta con el biomédico porque considera que la discapacidad no solo depende de las condiciones de la persona, sino de los apoyos que pueda brindar o no el entorno. Coincide este cambio de enfoque con la difusión en Perú del paradigma de la neurodiversidad ^28^, que nació en 1998 en Estados Unidos y que llegó a Latinoamérica casi una década después. Este paradigma considera que las CEA son parte de la variación natural en la especie humana, a nivel cognitivo y cerebral, y así como sugiere dificultades en algunas áreas, se asocia con fortalezas en otras.

En el año 2014 se Promulgó la Ley N.º 30150, Ley de protección de las personas con Trastorno del Espectro Autista ^29^, que tiene por objeto «establecer un régimen legal que fomente la detección y diagnóstico precoz, la intervención temprana, la protección de la salud, la educación integral, la capacitación profesional y la inserción laboral y social de las personas con trastorno del espectro autista». En 2019 se formuló el primer Plan Nacional para las personas con Trastorno del Espectro Autista (2019 - 2021) ^30^.

En los últimos años, el colectivo autista se ha hecho más visible socialmente, varias organizaciones realizan eventos de visibilización en los días representativos y algunos medios de comunicación difunden noticias relacionadas; así mismo se han dado muchos avances en intervención interdisciplinaria que permiten mejorar la calidad de vida de las personas con CEA; sin embargo, no es suficiente para avanzar respecto al reconocimiento y la defensa de los derechos de las personas con CEA.

En la Figura 2 se observa una línea de tiempo que sintetiza lo señalado sobre la historia del autismo en Perú; para evidenciar el contraste, se presenta información tanto en Perú como en el resto del mundo, sobre el uso de términos, los enfoques de intervención y los hitos, desde la década de 1910 a 2010. La variación de los términos asociados al autismo a lo largo del tiempo, desde «demencia precoz» hasta «autismo infantil» y «trastornos del espectro autista», se relacionan con la percepción que se ha tenido de la condición y su abordaje.

**Figura 2:** Línea del tiempo del autismo en Perú, en contraste con el resto del mundo.

Por otro lado, destaca el desfase constante entre el progreso del conocimiento sobre el autismo a nivel internacional y su integración en el contexto peruano. Esto se ve reflejado en la disparidad en la disponibilidad de servicios, acceso a la atención especializada en salud y educación, e investigación. Este contraste resalta la necesidad de un enfoque renovado en el desarrollo e implementación de estrategias específicas para abordar las necesidades de las personas con CEA en el contexto peruano.

A pesar de los avances significativos en la detección temprana, el diagnóstico, y la intervención desde la década de 1980 y particularmente en el siglo XXI, aún existen desafíos considerables en Perú en cuanto a la comprensión amplia de las CEA, la disponibilidad y acceso a servicios especializados, y la integración de estas personas en la sociedad. Actualmente, el Perú enfrenta aún muchos retos en torno a la comprensión de las CEA, el diagnóstico (especialmente el diagnóstico temprano), la epidemiología, la legislación, el manejo e investigación. Entender y reflexionar sobre la historia del autismo en Perú nos permite tener un marco de referencia para enfrentar estos desafíos.

Las CEA representan un desafío global, dada la prevalencia cercana al 1%, su estudio ha cobrado una importancia creciente a nivel mundial. Sin embargo, en el contexto peruano, se ha observado una notable carencia de investigaciones históricas específicas sobre el autismo. Este artículo surge como respuesta a esta brecha de investigación, destacando la evolución de los términos asociados al autismo a lo largo del tiempo, desde la denominación de «demencia precoz» en 1938, pasando por «esquizofrenia infantil» en 1944, hasta llegar a «autismo infantil» en 1959, y la posterior extensión del diagnóstico en la década de 1980, así como los avances y desafíos en el siglo XXI. Estos hallazgos no solo son relevantes desde una perspectiva histórica, sino también tienen importantes implicancias para la concienciación pública, el desarrollo de políticas de salud y la implementación de servicios especializados para abordar eficazmente el autismo en el contexto específico de Perú.
